# Reversible Thrombocytopenia after Gabapentin in an HIV-Positive Patient

**DOI:** 10.1155/2018/5927065

**Published:** 2018-01-04

**Authors:** Mohammed Basith, Andrew Francis, Alfredo Bellon

**Affiliations:** Department of Psychiatry, Penn State Milton S. Hershey Medical Center, Hershey, PA, USA

## Abstract

Gabapentin has become increasingly used in psychiatric practice specifically for anxiety disorders. Even though gabapentin is not approved by the US Food and Drug Administration to treat anxiety, physicians sometimes use it as an alternative to benzodiazepines in patients with a history of substance abuse. Gabapentin is also prescribed when individuals are at risk of thrombocytopenia which is not considered a side effect. Among patients at risk of thrombocytopenia are those positive for human immunodeficiency virus (HIV). Here we present a case of an HIV-positive man who presented for inpatient psychiatric care with severe anxiety and a history of alcohol and benzodiazepine abuse. In this patient, gabapentin worsened thrombocytopenia after repeated exposure to this medication. We suggest caution when considering gabapentin for patients with preexisting low platelet counts, as there seems to be a risk for worsening thrombocytopenia with this antiepileptic in the presence of HIV infection.

## 1. Introduction

Gabapentin is approved by the US Food and Drug Administration (FDA) for treatment of partial seizures with and without secondary generalization, as well as for postherpetic neuralgia, but not for psychiatric disorders [1]. Nonetheless, given the therapeutic value of other anticonvulsants, gabapentin has become increasingly studied and used in psychiatric practice specifically for anxiety disorders [2–4]. Relatively limited side effects (i.e., can lead to sedation and ataxia among others and it also requires dose adjustment for patients with renal impairment), few drug interactions, low addictive potential, and lack of monitoring requirements make gabapentin a favorable alternative to other antianxiety medications such as benzodiazepines [2, 3]. Thrombocytopenia is not considered a proven side effect of gabapentin, as only one previous report has suggested a relationship [5]. Although gabapentin is seldom used in treatment of seizures due to discovery of newer anticonvulsants, it had previously been used as an alternative to valproate and carbamazepine in treating seizures when patients were at risk of low platelets [6, 7]. Patients at risk of thrombocytopenia include individuals positive for human immunodeficiency virus (HIV). In this population, thrombocytopenia is well known as a chronic and often asymptomatic clinical finding independent of medication [8]. To our knowledge, gabapentin-induced thrombocytopenia has not been reported in the context of HIV infection. Therefore, here we report a case of an HIV-positive 36-year-old man with repeated thrombocytopenia linked to gabapentin.

## 2. Case Presentation

The patient was a 36-year-old Caucasian male with a history of recurrent major depressive disorder, alcohol use disorder, benzodiazepine use disorder, and no other known medical history. At the time of admission to our inpatient psychiatric unit, his platelet count was 88k [normal range 129–366k/uL] and his white blood cell count was 4.7 [normal range 3.9–9.5k/uL]. Other routine laboratory studies including basic metabolic panel, complete blood count, and liver function tests were within normal limits except a slightly low total bilirubin of 0.2 [normal range 0.3–1.0 mg/dL] and slightly low total protein of 6.0 [normal range 6.4–8.9 g/dL]. Of note, his creatinine was within normal limits and his estimated creatinine clearance was also normal. He was not aware of the low platelet count and no prior laboratory studies were available. His platelet count was monitored serially. He was also tested for HIV due to the unexplained low platelet count. He had never taken gabapentin. He was started on gabapentin at 300 mg/day and venlafaxine at 37.5 mg/day, which was titrated up to 150 mg/day and then held constant. His other medications included folic acid and thiamine during this period. He was not on other medications and did not have any allergies to any medications. His last alcohol use was at least five days prior to initiation of gabapentin and he noted intermittent, but not recent, benzodiazepine use.

**Figure 1:**
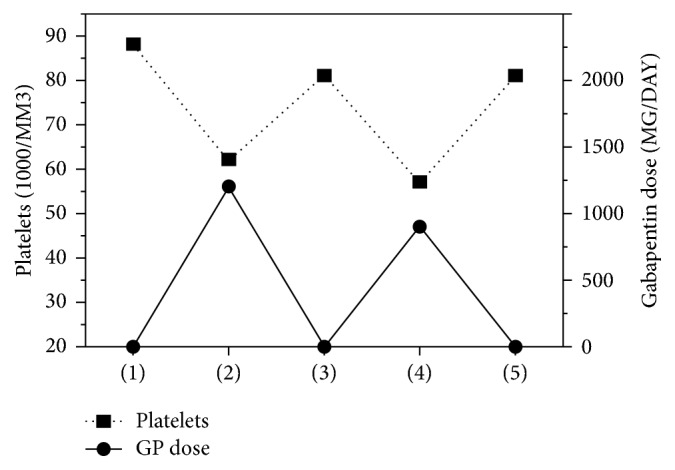
*Platelet Levels and Gabapentin Doses*. Data graphed over the same time course, specified as (1) initial value, (2) after first titration of gabapentin, (3) after gabapentin discontinued, (4) after second titration of gabapentin (restarted due to efficacy and consideration that thrombocytopenia might be secondary to HIV-induced thrombocytopenia rather than medication), and (5) after gabapentin discontinued again.

Gabapentin was titrated up to 1200 mg/day and was found to be effective for anxiety, but, within six days, the patient's platelet count dropped to 61k/uL (Figure 1), and gabapentin was discontinued while other medications remained the same. His platelets rose to 81k/uL within four days of discontinuing gabapentin. During this period, the HIV test result returned positive for HIV-1. The patient continued to be anxious, so gabapentin was restarted due to its efficacy and consideration that thrombocytopenia might be related to HIV status. Again, within three days of restarting gabapentin at 900 mg/day and with no other changes in medication, the patient's platelet count dropped to 57k/uL. Gabapentin was discontinued again and, within two days, the platelet count increased back to 81k/uL (Figure 1).

## 3. Discussion

The repeated exposure with a time-linked and reversible decrease in platelets indicates an association between gabapentin and thrombocytopenia in this patient with HIV. It is possible that HIV-induced thrombocytopenia may have a sensitizing effect on the action of gabapentin. It is also possible that venlafaxine could have an effect on decreasing platelets, although the dose was titrated up to 150 mg/day but then held constant during the titrations of gabapentin. Another possibility is that chronic alcohol use could have an effect on low platelets, but the patient was not drinking or using substances for at least five days prior to starting these titrations and he was under inpatient care during this time. Based on the Naranjo Adverse Drug Reaction Probability Scale, the changes which occurred after gabapentin was initiated were seen again on reinitiation, had limited alternate causes (these seem to be unlikely given the time-linked change), seemed to be worse at a higher dose, and were objectively measured by platelet count [9]. Scoring on the Naranjo scale therefore suggests probable to definite likelihood of an effect related to gabapentin. These observations suggest caution when considering gabapentin for patients with preexisting low platelet counts and suggest a potential for thrombocytopenia with gabapentin especially in the presence of HIV infection.
